# Role of high-dose salvage radiotherapy for oligometastases of the localised abdominal/pelvic lymph nodes: a retrospective study

**DOI:** 10.1186/s12885-020-07033-7

**Published:** 2020-06-09

**Authors:** Makoto Ito, Takeshi Kodaira, Yutaro Koide, Takahito Okuda, Shinichiro Mizumatsu, Yukihiko Oshima, Arisa Takeuchi, Toshie Mori, Souichirou Abe, Ayumi Asai, Kojiro Suzuki

**Affiliations:** 1 Department of Radiology, Aichi Medical University Hospital, 1-1 Yazako-Karimata, Nagakute, Aichi 480-1195 Japan; 2 Department of Radiation Oncology, Aichi Cancer Center Hospital, 1-1 Kanokoden, Chikusa-ku, Nagoya, Aichi 484-8681 Japan; 3 Department of Radiation Oncology, Toyota Memorial Hospital, 1-1-1 Heiwa-cho, Toyota, Aichi 471-8513 Japan; 4 Department of CyberKnife Center, Aoyama Hospital, 100-1 Kozakai-tyo-doji, Toyokawa, Aichi 441-0195 Japan

**Keywords:** Oligometastasis, Oligo-recurrence, Radiotherapy, Stereotactic body radiotherapy (SBRT), Lymph node

## Abstract

**Background:**

Abdominal/pelvic lymph node (LN) oligometastasis, a pattern of treatment failure, is observed occasionally, and radiotherapy may work as salvage therapy. The optimal prescription dose, however, is yet to be determined. This study assessed the efficacy of high-dose radiotherapy.

**Methods:**

The medical records of 113 patients at 4 institutes were retrospectively analysed who had 1 to 5 abdominal/pelvic LN oligometastases and were treated with definitive radiotherapy between 2008 and 2018. The exclusion criteria included non-epithelial tumours, uncontrolled primary lesions, palliative intent, and re-irradiation. The prescription dose was evaluated by using the equivalent dose in 2 Gy fractions (EQD2). Patients receiving EQD2 ≥ 60 Gy were placed into the high-dose group, and the remaining others the low-dose group. Kaplan-Meier analyses were performed to evaluate overall survival (OS), local control (LC), and progression-free survival (PFS). Univariate log-rank and multivariate Cox proportional hazards model analyses were performed to explore predictive factors. Adverse events were compared between the high-dose and low-dose groups.

**Results:**

The primary tumour sites included the colorectum (*n* = 28), uterine cervix (*n* = 27), endometrium (*n* = 15), and ovaries (*n* = 10). The rate of 2-year OS was 63.1%, that of LC 59.7%, and that of PFS 19.4%. On multivariate analyses, OS were significantly associated with solitary oligometastasis (hazard ratio [HR]: 0.48, *p* = 0.02), LC with high-dose radiotherapy (HR: 0.93, *p* < 0.001), and PFS with long disease-free interval (HR: 0.59, *p* = 0.01). Whereas high-dose radiotherapy did not significantly improve 2-year OS in the entire cohort (74.8% in the high-dose vs. 52.7% in the low-dose; *p* = 0.08), it did in the subgroup of solitary oligometastasis (88.8% in the high-dose vs. 56.3% in the low-dose; *p* = 0.009). As for Late grade ≥ 3 adverse event, ileus was observed in 7 patients (6%) and gastrointestinal bleeding in 4 (4%). No significant association between the irradiation dose and adverse event incidence was found.

**Conclusions:**

As salvage therapy, high-dose radiotherapy was recommendable for oligometastasis in the abdominal/pelvic LNs. For solitary oligometastasis, LC and OS were significantly better in the high-dose group.

## Background

The prognosis for recurrent cancer is generally poor, and distant metastasis is especially difficult to cure. Therefore, the standard treatment of metastases is systemic chemotherapy; however, local therapy is often used to treat a small number of recurrent tumours. In 1995, oligometastases were defined as metastatic disease confined to a limited number of sites that were amenable to potentially curable local therapy such as surgery or radiotherapy [1].

Abdominal/pelvic lymph node (LN) oligometastasis is a pattern of failure that is observed occasionally, and it is usually unresectable [2, 3]. Although radiotherapy may be the treatment choice for salvage therapy, treatment is very difficult because abdominal/pelvic LNs move randomly owing to the effect of breathing and being located close to the gastrointestinal tract. Therefore, only a few studies have evaluated the use of radiotherapy for abdominal/pelvic LN oligometastasis [4, 5]. In particular, the optimal prescription dose for radiotherapy has not yet been clarified. The important issue is that most of them analysed cases together regardless of whether or not lesions other than LN metastasis were tightly controlled [6]. Therefore the discussions focused on local control (LC), and the survival benefits of controlling LNs were not clear. In addition, prescription doses were not uniform [6]. This makes it more difficult to determine the optimal dose for survival.

Along with the increasing use of high-precision irradiation technology, reports regarding the use of high-dose radiotherapy for oligometastasis are also increasing [7, 8]. Recently, the SABR-COMET trial, a randomised phase 2 open-label trial, compared radiotherapy and standard of care palliative treatment for oligometastasis [9]. According to the trial, stereotactic ablative radiotherapy (SABR) was associated with improved overall survival (OS). However, treatment-related death was observed in 4.5% of patients; therefore, researchers concluded that high-dose radiotherapy should be adapted for those who would show a survival benefit. In addition, as various irradiation regions were included in the trial, further study is required to evaluate the balance between the risks and benefits of localised irradiation. Moreover, it is important to clarify the role of high-dose radiotherapy especially in locations where radiotherapy is difficult to perform, such as abdominal/pelvic LNs.

Therefore, in this study, we retrospectively reviewed the data of patients with oligometastases in the abdominal/pelvic LNs and investigated the optimal method of salvage radiotherapy. Moreover, we assessed the survival benefit of high-dose radiotherapy.

## Methods

### Ethics approval and study design

This retrospective observational study was approved by ethics committee, Aichi Medical University School of Medicine in Japan (application number 2018-H211). This study was also examined and approved by Aichi Cancer Center Hospital, Toyota Memorial Hospital, and Aoyama Hospital, Japan. The study was conducted in accordance with the tenets of the Declaration of Helsinki and its subsequent amendments. Written informed consent was obtained from all patients.

### Patients

Between January 2008 and April 2018, the medical records of patients treated with radiation to their abdominal/pelvic LN with a definitive intent were retrospectively reviewed from 4 institutes. In this study, patients aged 20 to 85 years who developed localised abdominal/pelvic oligometastases in 1–5 LNs after initial treatment were included. The exclusion criteria were as follows: patients with non-epithelial tumours, those with uncontrolled primary lesions, patients treated with palliative intent, those with a short follow-up (< 3 months), and those who received re-irradiation. Finally, a total of 113 patients were included.

The initial treatment of the primary disease was either surgery alone (*n* = 101) or definitive radiotherapy (*n* = 12). Before undergoing salvage radiotherapy, all the patients underwent whole-body computed tomography (CT) and/or positron emission tomography-CT (PET-CT) to confirm that there were no oligometastases other than those in the abdominal/pelvic LNs. Owing to the presence of oligometastases after initial treatment, 91 patients underwent chemotherapy and 33 underwent concurrent chemotherapy and salvage radiotherapy.

### Radiotherapy

A linear accelerator or CyberKnife (CK) was used for salvage radiotherapy. Patients were immobilised in the supine position, and a CT scan with a 1–5-mm slice thickness was obtained for treatment planning. For high-precision radiotherapy, such as intensity-modulated radiotherapy (IMRT) and stereotactic body radiotherapy (SBRT), an external vacuum-type body mould and/or a thermoplastic body mask were used for precise fixation. To avoid large displacement of the gastrointestinal tract during daily treatment, planning CT was performed with the patient in a fasting state, especially when the target was near the stomach. The clinical target volume (CTV) was defined as the gross tumour volume with a 0–0.5-cm margin considering microscopic disease and the anatomical structure. The planning target volume (PTV) was defined as the CTV with a 0.3–1.0-cm margin, considering the internal motion and the setting error in each institution. Moreover, the PTV margin was suitably adjusted to protect organs at risk (OARs). While using the linear accelerators, the abdominal/pelvic LN region was divided into 4 regions: para-aortic, iliac, presacral, and obturator. The nodal area that includes the gross tumour was defined as a prophylactic nodal area. Regarding iliac and obturator regions, the side of the tumour was delineated. In cases of 2 oligometastases or more, prophylactic nodal areas were made smaller so that the irradiation area would not be too large. Prophylactic nodal areas were expanded by 0.5 cm considering setup errors. The main OARs were evaluated by using the equivalent dose in 2 Gy fractions (EQD2) at α/β = 3, including the stomach, small bowel, large bowel, kidneys, and spinal cord. For 3-dimensional conformal radiotherapy (3DCRT), dose constraints were defined as follows: the maximum point dose was 52 Gy to the stomach and small bowel, 62 Gy to the large bowel, and 50 Gy to the spinal cord. For high-precision radiotherapy the dose constraints were defined as follows: the stomach and small bowel received a point dose of 54 Gy (V54) for < 3 cm^3^, the large bowel V64 for < 3 cm^3^, and the kidneys a V50 of < 33%. The maximum point dose to the spinal cord was < 52 Gy.

#### Linear accelerator

Sixty-one patients were treated with a linear accelerator. Both the target volume and normal organ structures were contoured using treatment planning systems (either XiO, Electa, CMS, St Louis, MO, USA or Eclipse, Varian Medical System, Palo Alto, CA, USA). All the patients were treated with 10 MV photons, using the 3DCRT or IMRT technique. Patients treated with 3DCRT (*n* = 47) were prescribed 39.6–70 Gy in 15–37 fractions to the PTV isocenter; the median dose per fraction was 2 Gy (range, 1.8–3 Gy) and the median EQD2 was 50 Gy. In contrast, patients treated with IMRT (*n* = 14) received 45–70 Gy in 15–35 fractions, where the PTV was covered with a 95% dose; the median dose per fraction was 2 Gy (range, 1.8–3.5 Gy) and the median EQD2 was 61.4 Gy. More detailed data such as treatment modality and dose fractionation are shown in Supplementary Table 1. The prophylactic nodal areas received almost 70–80% of the PTV dose in both 3DCRT and IMRT. Either sequential boost or simultaneous integrated boost technics were used in patients who received prophylactic nodal irradiation with IMRT.

#### CyberKnife

Fifty-two patients were treated with CyberKnife. All SBRT patients were treated with CyberKnife. Ten patients were treated with less than 4 Gy per fraction, and they were classified as IMRT. CK G4 and M6 (Accuray Inc., Sunnyvale CA, USA) and B were used for SBRT. In addition to using the IRIS variable collimator, the InCise multileaf collimator was used and contributed to reduction in the treatment time for CK-M6. Generally, the tumour was followed-up by using the fiducial-less tracking capability of CK under free breathing. Using a 6-MV photon beam, all treatment plans were generated using the Multiplan treatment planning software (Accuray Inc., Sunnyvale, CA, USA). The dose was prescribed for covering the PTV, ranging from 21 to 60 Gy, and it was fractionated 2 to 30 times with a 60 to 90% isodose line; the median dose per fraction was 6 Gy (range, 2–13.5 Gy) and the median EQD2 was 63.5 Gy.

### Data collection and statistical analyses

As the fractionation schedules and dose evaluation method were not standardised, the dose-volume histograms of all the cases were confirmed. Then, the prescription dose of all the cases was re-evaluated by using the EQD2 (D50%) with α/β = 10. The median EQD2 for all the patients was 59.7 Gy (range, 40.3–101.4 Gy); using the median value as the cut-off, the high-dose group was defined as patients with EQD2 ≥ 60 Gy and the low-dose group was defined as those with EQD2 <  60 Gy.

According to the Response Evaluation Criteria in Solid Tumors, the initial tumour response was evaluated based on CT scans [10]. Complete response was defined as a reduction of the lymph node to < 10 mm in the short axis. Partial response was defined as at least a 30% decrease from the baseline. Progressive disease was at least a 20% increase, taking as reference the smallest state. Stable disease was defined as having neither sufficient shrinkage to qualify for partial response nor sufficient increase to qualify for progressive disease. The first follow-up visit was scheduled within 1–4.5 months (median, 2.2 months) after the end of treatment. The disease-free interval (DFI) was defined as the time from the last treatment to salvage radiotherapy. The primary endpoint was the OS. The secondary endpoints included LC, progression-free survival (PFS), and adverse events. OS was measured from the start date of salvage radiotherapy to the date of the last follow-up or death from any cause. Local control (LC) was defined as progression in the target LN as evaluated on CT or PET-CT images. Progression-free survival (PFS) was calculated from the start date of salvage radiotherapy until the date of disease progression or death from any cause.

The OS, LC, and PFS rates were estimated using the Kaplan-Meier method [11]. Log-rank tests were used to compare the estimates of subgroups on univariate analysis. The Cox proportional hazards model was used for multivariate analysis. *P*-values < 0.05 were statistically significant. Factors that showed a difference with *p* < 0.1 on univariate analysis were entered into the multivariate analysis. Adverse events were graded according to the National Cancer Institute Common Toxicity Criteria for Adverse Events (version 5.0), and grade ≥ 3 events were counted. As supplementary information, events after re-irradiation were collected as secondary adverse events, separately from the primary adverse events. Re-irradiation here does not refer to the second radiotherapy in the same area as the initial treatment, which was excluded in the study, but to the one performed for oligometastasis of the abdominal/pelvic LNs. All statistical analyses were performed with EZR version 1.33 (Saitama Medical Center, Jichi Medical University, Saitama, Japan), based on the R and R commander [12].

## Results

### Patient characteristics

The median follow-up period for all the included 113 patients was 17.8 months (range, 3.7–109.8 months). At the time of analysis, 46 patients had died. The median follow-up time for the remaining surviving patients was 23.7 months (range, 4.0–109.8 months). The patient characteristics are listed in Table 1 and the relationship between the lymph node site and the number of lesions is shown in Supplementary Table 2. Regarding the primary site, the number of cases of colorectal and ovarian cancer was high in the high-dose group, while the number of cases of uterine cervix and endometrium cancer was high in the low-dose group. There were no patients with prostate cancer by chance. Significantly more patients were treated with SBRT and IMRT in the high-dose group. Prophylactic nodal irradiation and concurrent chemotherapy were often performed with 3DCRT. The median EQD2 to the PTV in the high-dose group, the low-dose group, and all patients was 66.6 Gy (range, 60.4–101.4 Gy), 50 Gy (range, 40.3–59.8 Gy), and 59.7 Gy (range, 40.3–101.4 Gy), respectively.

**Table 1:** Patient characteristics

Characteristic		High-dose group (*n* = 55)	Low-dose group(*n* = 58)	All cases (*n* = 113)
Age (years)	Median	67	62.5	65
	Range	49–83	36–83	36–83
Gender	Male	24	21	45
	Female	31	37	68
Performance status	0	35	33	68
	1	18	24	42
	2	2	1	3
Primary site	Colorectum	20	8	28
	Uterine cervix	9	18	27
	Endometrium	5	10	15
	Ovary	8	2	10
	Urothelial	4	4	8
	Others	9	16	25
Histopathology	Adenocarcinoma	41	31	72
	Squamous cell carcinoma	10	23	33
	Others	4	4	8
Initial category	T-category 1:2:3:4	13:20:18:4	18:13:19:8	31:33:37:12
	N-category positive	32	32	64
Duration from initial diagnosis (months)	Median	25.4	22.4	24.1
	Range	2.9–102.4	1.9–100.4	1.9–102.4
DFI (months)	Median	8.7	8.3	8.5
	Range	0.6–64.4	0.5–86.6	0.5–86.6
Radiation therapy method	3DCRT	12	35	47
	SBRT&IMRT	43	23	66
LN site	Para-aortic	29	43	72
	Iliac	8	10	18
	Presacral	9	3	12
	Obturator	9	2	11
Extra-regional LN for primary site	no	37	28	65
	yes	18	30	48
Number of lymph node	1	33	28	61
	2–5	22	30	52
LN size (cm)	Median	2	2	2
	Range	1–5.5	1–5	1–5.5
Prophylactic nodal irradiation	no	40	29	69
	yes	15	29	44
Chemotherapy	no	15	7	22
	yes	40	51	91
Concurrent chemotherapy	no	43	37	80
	yes	12	21	33
EQD2 (Gy)	Median	66.6	50	59.7
	Range	60.4–101.4	40.3–59.8	40.3–101.4
Follow-up time of entire group (months)	Median	20.2	15.9	17.8
	Range	5.8–84.7	3.7–109.8	3.7–109.8
Follow-up time in surviving patients (months)	Median	23	24.2	23.7
	Range	7.6–84.7	4.0–109.8	4.0–109.8

*n* number of patients, *LN* lymph node, *DFI* disease-free interval, *3DCRT* 3-dimentional conformal radiation therapy, *SBRT* Stereotactic Body Radiation Therapy, *IMRT* intensity-modulated radiation therapy, *EQD2* equivalent dose in 2 Gy fraction

### Treatment outcomes

The 2-year OS, LC, and PFS rates were 63.1% (95% CI: 52.1–72.2%), 59.7% (95% CI: 48.8–69.0%), and 19.4% (95% CI: 12.2–27.8%), respectively. A total of 67 patients (59.3%) were alive at the time of analysis: 36 were alive without disease and 31 were alive with disease. Only 3 patients died of other causes: pneumonia (*n* = 2) and owing to the adverse effects of a steroid pulse for myelitis (*n* = 1). Regarding the initial response in the entire group (*n* = 113), 23 patients showed a complete response (CR), 66 showed a partial response (PR), 19 had stable disease, and 5 had progressive disease. There was no significant difference in the CR/PR rate between the high-dose and low-dose groups (80.0% vs. 77.6%, *p* = 0.82).

The results of univariate analysis for the OS, LC, and PFS rates are shown in Table 2. Neither the primary site nor the pathological type significantly contributed to any endpoint. A longer DFI (≥ 8.5 months) was associated with significantly better 2-year OS (72.5% vs. 53.4%, *p* = 0.04) and PFS (24.9% vs. 13.5%, *p* = 0.01). The number of LNs was a prognostic factor, and solitary oligometastasis showed favourable OS (73.3% vs. 51.3%, *p* = 0.01) and LC (70.4% vs. 47.8%, *p* = 0.01) compared with multiple metastasis. In contrast, the size of the LNs did not show apparent correlation with any parameters. Although the high-dose group had significantly better LC than the low-dose group (74.9% vs. 45.2%, *p* < 0.001), there was no significant difference in OS between the groups.

**Table 2:** Univariate analysis of prognostic factors of each endpoint

Factor		2y-OS		2y-LC		2y-PFS	
		%	*P*	%	*P*	%	*P*
Age (years)	< 66 (*n* = 57)	56.6	0.21	50.8	0.04	16.2	0.150
	≥ 66 (*n* = 56)	71.3		69.2		22.5	
Gender	Male (*n* = 45)	55.1	0.11	59.1	0.42	21.1	0.97
	Female (*n* = 68)	68.2		60.3		17.8	
Performance status	0 (*n* = 68)	69.2	0.49	61.6	0.97	19.1	0.93
	1–2 (*n* = 45)	54.5		56.7		19.9	
Primary site	Colorectum	73.5	0.49	64.1	0.79	21.9	0.96
	Uterine cervix	88.4		60.7		25.8	
	Endometrium	54.4		59.3		23.3	
Pathological type	Adenocarcinoma	66.1	0.86	59.7	0.37	19.4	0.76
	Squamous cell carcinoma	67.2		60.4		23.5	
Initial T-category	T1–2 (*n* = 64)	68.7	0.07	64.2	0.61	22.9	0.69
	T3–4 (*n* = 49)	56.0		54.7		16.2	
Initial N-category	N negative (*n* = 49)	66.6	0.09	64.2	0.72	23.8	0.61
	N positive (*n* = 64)	60.2		56.1		16.6	
Duration from initial diagnosis (months)	< 24.1 (*n* = 57)	60.9	0.94	63.8	0.43	20.7	0.59
	≥ 24.1 (*n* = 56)	63.9		54.9		18.0	
DFI (months)	< 8.5 (*n* = 57)	53.4	0.04	52.4	0.30	13.5	0.01
	≥ 8.5 (*n* = 56)	72.5		66.0		24.9	
Radiation therapy method	3DCRT (*n* = 47)	56.1	0.49	50.9	0.01	26.0	0.82
	IMRT&SBRT (*n* = 66)	67.9		64.9		14.5	
Extra-regional LN for primary site	no (*n* = 65)	65.5	0.07	60.6	0.92	21.9	0.45
	yes (*n* = 48)	59.7		57.7		15.3	
Number of LN	1 (*n* = 61)	73.3	0.01	70.4	0.01	25.1	0.08
	2–5 (*n* = 52)	51.3		47.8		12.9	
LN size (cm)	< 2 (*n* = 62)	71.6	0.17	66.0	0.06	18.1	0.68
	≥ 2 (*n* = 51)	52.0		52.4		20.7	
Prophylactic nodal irradiation	no (*n* = 69)	63.7	0.69	63.3	0.41	15.8	0.80
	yes (*n* = 44)	61.4		54.9		28.2	
Concurrent chemotherapy	no (*n* = 80)	63.5	0.92	58.8	0.69	11.0	0.20
	yes (*n* = 33)	59.5		60.8		36.1	
EQD2 (Gy)	< 60 (*n* = 58)	52.7	0.08	45.2	< 0.001	21.1	0.87
	≥ 60 (*n* = 55)	74.8		74.9		17.5	

*n* number of patients, *OS* overall survival rate, *LC* local control rate, *PFS* progression-free survival rate, *DFI* disease-free interval, *3DCRT* 3-dimentional conformal radiation therapy, *IMRT* intensity-modulated radiation therapy, *SBRT* Stereotactic Body Radiation Therapy, *LN* lymph node, *EQD2* equivalent dose in 2 Gy fraction

On multivariate analyses, solitary oligometastasis was significantly correlated with OS (HR: 0.48, 95% CI: 0.27–0.87, *p* = 0.02); high-dose radiotherapy with LC (HR: 0.93, 95% CI: 0.90–0.96, *p* < 0.001); and long DFI with PFS (HR: 0.59, 95% CI: 0.39–0.90, *p* = 0.01) (Table 3).

**Table 3:** Multivariate analysis of each endpoint

Factor		OS			LC			PFS		
		HR	95% CI	*P*	HR	95% CI	*P*	HR	95% CI	*P*
Age (years)	< 66 vs ≥ 66				0.99	(0.97–1.02)	0.69			
Initial T-category	T1–2 vs T3–4	1.57	(0.86–2.83)	0.14						
Initial N-category	N negative vs N positive	1.33	(0.69–2.53)	0.39						
DFI (months)	< 8.5 vs ≥ 8.5	0.99	(0.99–1.01)	0.11				0.59	(0.39–0.90)	0.01
Radiation therapy method	3DCRT vs IMRT&SBRT				0.95	(0.46–1.97)	0.89			
Extra-regional LN for primary site	no vs yes	0.87	(0.45–1.67)	0.67						
Number of LN	1 vs 2–5	0.48	(0.27–0.87)	0.02	0.56	(0.30–1.03)	0.06	0.71	(0.47–1.08)	0.11
LN size (cm)	< 2 vs ≥ 2				1.52	(0.83–2.80)	0.18			
EQD2 (Gy)	< 60 vs ≥ 60	0.97	(0.95–1.01)	0.09	0.93	(0.90–0.96)	< 0.001			

*HR* hazard ratio, *CI* confidence interval, *OS* overall survival rate, *LC* local control rate, *PFS* progression-free survival rate, *DFI* disease-free interval, *3DCRT* 3-dimentional conformal radiation therapy, *IMRT* intensity-modulated radiation therapy, *SBRT* Stereotactic Body Radiation Therapy, *LN* lymph node, *EQD2* equivalent dose in 2 Gy fraction

### Subgroup analysis for solitary oligometastasis

**Fig. 1:**
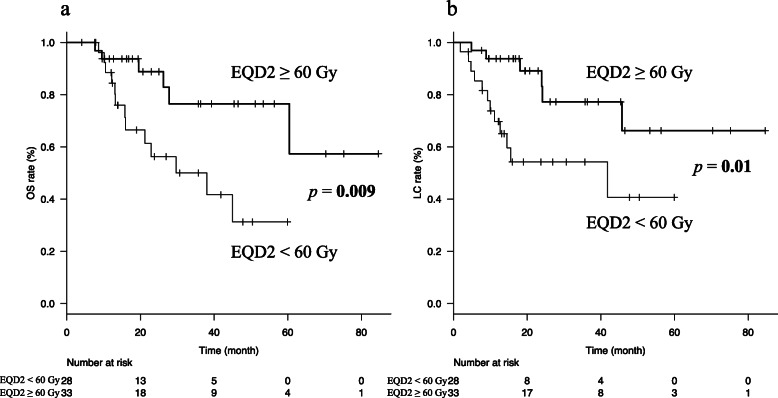
Kaplan-Meier curves in the solitary oligometastasis groups. Kaplan-Meier curves for OS (**a**) and LC (**b**) in the solitary oligometastasis groups separated by EQD2. OS, overall survival; LC, local control; EQD2, equivalent dose in 2 Gy fractions

High-dose radiotherapy did not significantly improve the 2-year OS on analysis of the entire cohort. However, on the subgroup analysis, solitary oligometastasis, as compared with multiple oligometastases, was a significantly favourable factor for 2-year OS (88.8% vs. 56.3%, *p* = 0.009, Fig. 1a) as well as for 2-year LC (83.2% vs. 54.2%, *p* = 0.01, Fig. 1b). The characteristics of patients with only solitary oligometastasis are shown in Supplementary Table 3. No significant bias in the important background factors seemed to contribute to OS on univariate analysis. Furthermore, another subgroup analysis using more homogeneous patients was performed. The analysis included the patients who only had a solitary lymph node metastasis in the para-aortic site. The patients were excluded if the size of the LN was larger than 3 cm and/or they received greater than 10 Gy per fraction. The remaining 35 patients were placed into a high-dose group of 17 patients or a low-dose group of 18 patients. Though the difference was not significant, the high-dose radiotherapy group showed a favourable outcome for 2-year OS (93.8% vs. 56.5%, *p* = 0.09) as well as for 2-year LC (85.2% vs. 61.6%, *p* = 0.10).

### Adverse events

No significant difference was found in the incidence of grade ≥ 3 adverse events between the high-dose and low-dose groups. Of acute primary adverse events, grade 3 diarrhoea, was observed in 3 patients (3%), stomach-ache in 1 patient (1%), and loss of appetite in 1 patient (1%). No grade ≥ 4 primary adverse events or grade ≥ 3 secondary adverse events were observed.

As for late primary adverse events, grade 3 ileus developed in 7 patients (6%), with 4 (3%) in the high-dose group and 3 (3%) in the low-dose group. Of the 7 patients, 6 (86%) received prophylactic nodal irradiation. Three of them (43%) were diagnosed with ileus and underwent elective bowel resection or synectenterotomy. Although 2 of them (29%) received re-irradiation, ileus as a secondary adverse event was not observed. Grade ≥ 3 gastrointestinal bleeding was observed in 4 patients (4%): in two patients as a primary adverse event, and in the other two as secondary adverse events. The details of these 4 patients are shown in Table 4. Of the 4 patients, 2 showed grade 3 gastrointestinal bleeding and both had received re-irradiation, while the remaining 2 patients had grade 4 and grade 5 gastrointestinal bleeding, respectively, with 1 patient each in the high-dose and low-dose groups. Although both these patients were identified as having PR as the initial response, disease progression was again observed during follow-up. Chemotherapy or re-irradiation could not be administered again, and best supportive care was performed. Accordingly, the cause of serious gastrointestinal bleeding for these 2 patients was clinically interpreted to be tumour progression instead of treatment-related.

**Table 4:** Details of patients with Grade ≥ 3 gastrointestinal bleeding

N	Technique	Prophylactic nodal irradiation	Time from 1st RT to re-irradiation (months)	Time from 1st/2nd RT to bleeding (months)	^a^EQD2 prescribed from 1st/cumulative RT (Gy)	Location of bleeding	Tumor control at bleeding	Grade	Therapy for bleeding
1	3DCRT	No	NA	13.2	61.8	large bowel	No	4	Emergency colonoscopy
2	3DCRT	Yes	15.2	34.1/18.9	44.3/82.3	small bowel	Yes	3	Blood transfusion
3	3DCRT	No	9.6	16.8/7.2	38.2/74.8	large bowel	Yes	3	Blood transfusion
4	SBRT	No	NA	9.6	28.2	small bowel	No	5	Best supportive care

*N* patient number, *3DCRT* 3-dimentional conformal radiation therapy, *SBRT* Stereotactic Body Radiotherapy, *1st* First, *2nd* second, *EQD2* equivalent dose in 2 Gy fraction, *NA* not applicable

^a^Prescribed EQD2 for location of bleeding was evaluated with α/β = 3.Cumulative doses were estimated by fusing the first and second dose distributions

Re-irradiation was performed for a total of 6 patients after salvage radiotherapy: 2 patients in the high-dose group and the remaining 4 in the low-dose group. As described above, 2 of the 6 patients (33%) presented with grade 3 late secondary adverse events after re-irradiation; both of them were in the high-dose group.

## Discussion

In the current retrospective study of 113 patients with abdominal/pelvic LNs recurrent cancer and oligometastasis from 4 institutions, the 2-year OS, LC, and PFS rates were 63.1, 59.7, and 19.4%, respectively. Among grade ≥ 3 late adverse events, ileus was observed in 7 patients (6%) and gastrointestinal bleeding in 4 (4%). The efficacy and safety were similar to those observed in previous studies [4, 5, 13–15].

In the current study, the high-dose group (*n* = 55) had significantly improved 2-year LC rates (74.9% in the high-dose group vs. 45.2% in the low-dose group; *p* < 0.001); however, improvement in 2-year OS did not reach statistical difference (74.8% in the high-dose group vs. 52.7% in the low-dose group; *p* = 0.08). A reason for this might be that our cohort included patients with a poor prognosis, which is a bias accompanying the retrospective nature of the study. One of the important prognostic factors is the condition of the primary lesion. Niibe et al. defined the concept of oligo-recurrence as the state in which patients with cancer have ≤5 metastatic or recurrent lesions with a controlled primary lesion; they showed better prognosis than metastatic cases with active primary lesions [16]. Accordingly, in the current study, the inclusion of only patients with oligometastases was insufficient for analysis. The effect of the tumour burden was observed in ≤5 LNs, and the number of oligometastases seems to be a very important prognostic factor. Oehler C et al. defined up to 3 lymph node recurrences as oligorecurrent and retrospectively analyzed SBRT outcomes for prostate cancer after radical prostatectomy [17]. In their study, the tumor size was scrutinized and hypotheses were established as predictors of biochemical control. Nakamura et al. performed a retrospective study of 76 patients with oligo-recurrent lesions in the lungs and liver treated with SBRT. In their study, a single metastatic lesion was a significant factor of good PFS (*p* = 0.008) and a favourable OS (*p* = 0.053) [18]. Similarly, in the current study, the presence of solitary oligometastasis was associated with significantly favourable OS (HR 0.48, 95% CI 0.27–0.87; *p* = 0.02). Furthermore, on subgroup analysis of solitary oligometastasis, high-dose radiotherapy significantly improved LC (*p* = 0.01) and OS (*p* = 0.009). Another subgroup analysis using more homogeneous patients did not reach statistical difference, probably due to the small number of patients. The results favoured the high-dose radiotherapy group and supported the outcomes of other analyses. From these results, patients with a solitary lymph node metastasis would have a favourable prognosis, and for such patients high-dose radiotherapy might work well on OS through improving LC.

The 2-year PFS was very poor (19.4%) regardless of the administration of prophylactic nodal irradiation. Although chemotherapy may contribute to tumour control, the precise advantage could not be determined because of the effect of bias in our study. Moreover, regarding the effectiveness of previous or adjuvant chemotherapy, there is no consensus till date [15, 18, 19]. Further individual analysis is required to clarify the efficacy of chemotherapy according to the primary disease. The DFI may be a good indicator of disease recurrence. Gandhidasan et al. reported 132 patients treated with SABR for oligometastasis [7]. In their analysis, a long DFI was significantly associated with favourable recovery from widespread disease (HR: 0.91, 95% CI: 0.82–1.01, *p* = 0.041). Similarly, in the current study, a long DFI was associated with significantly favourable PFS (HR: 0.59, 95% CI: 0.39–0.90, *p* = 0.01). Therefore, careful follow-up will be required especially in patients with a short DFI.

High-dose radiotherapy was as safe as low-dose radiotherapy, without any significant increase in the incidence of adverse events. However, re-irradiation especially after high-dose salvage radiotherapy might be a high-risk treatment. Of 6 patients who received re-irradiation, 2 patients (33%) presented with grade ≥ 3 late adverse events. Though this is a supplementary result, we propose that the execution of re-irradiation to the abdominal/pelvic LNs should be judged carefully. As the incidence of adverse events was high, we cannot recommend re-irradiation to the abdominal/pelvic LNs. In addition, prophylactic irradiation may be associated with the risk of ileus and its use should be considered carefully; however, as surgery is also associated with the risk of ileus, the use of prophylactic irradiation is not a major issue per se. Although a serious adverse event was observed infrequently in the current study, in the SABR-COMET trial the incidence of adverse events of grade ≥ 2 increased by 20, and 4.5% of patients in the SABR group experienced a treatment-related death [9]. Accordingly, we should determine the survival benefit of using salvage radiotherapy considering the risk of adverse events.

The current study was limited by its retrospective nature and multi-institution analysis. Although our cohort did not include patients with prostate cancer, which could have led to bias, we included patients with multiple cancer types. Thus, we cannot ignore the histology-specific differences in tumour biology. There was a bias in the patient background between the high- and low-dose groups, as well as the primary site and pathological type. In particular, the bias of the DFI and extra-regional nodes may have worked in favour of the high-dose groups. Furthermore, the treatment protocol was not constant, and the number of patients and the follow-up period were insufficient. The method of setting target volume and prescribing dose was not consistent. Especially the definition and application of prophylactic nodal irradiation were not unified. We re-evaluated with the D50 prescription, but the difference in the dose gradient from the prescription method cannot be completely eliminated. Since this study used data older than 10 years, it may be affected by treatment trends such as a hypofractionation. Accordingly, future trials should evaluate the treatment efficacy in a cohort of patients with the same tumour histology and using the same initial treatment protocol. Moreover, another limitation was the technical diversity. The margin for LNs was not standardized for all cases. In addition, the definition of the target volume varied depending on the prophylactic area. Although all the plans were reappraised by using EQD2, linear accelerators and the CK system have fundamentally different characteristics [20]. Three treatments, 3DCRT, IMRT, and SBRT, were standardized by EQD2 and evaluated together. Although a prescription dose greater than 10 Gy per fraction was present in only 1 case, it might not be appropriate to apply the EQD2 model to SBRT. Thus, the optimal irradiation dose should be evaluated using any one of the devices according to a single protocol.

## Conclusion

Salvage radiotherapy was a feasible and safe approach for oligometastasis of the abdominal/pelvic LNs, especially in patients treated with high-dose radiotherapy (EQD2 ≥ 60 Gy), as we observed excellent LC. For OS, the number of LNs was the most important factor, and solitary oligometastasis is an indicator of good prognosis. In patients with solitary oligometastasis, high-dose radiotherapy may play an important role in improving LC and OS. Accordingly, considering these promising results, a prospective study in a more homogeneous patient population is required to clarify the survival benefits of salvage high-dose radiotherapy.

## Supplementary information

**Additional file 1: Supplementary Table 1.** Treatment details for each patient. **Supplementary Table 2.** Number of lymph node metastases by site. **Supplementary Table 3.** Patient characteristics in the solitary oligometastasis group.

## Data Availability

The datasets used and/or analysed during the current study are available from the corresponding author on reasonable request.

## Abbreviations

3DCRT: 3-dimensional conformal radiotherapy
CI: Confidence interval
CK: CyberKnife
CR: Complete response
CT: Computed tomography
CTV: Clinical target volume
DFI: Disease-free interval
EQD2: Equivalent dose in 2 Gy fractions
HR: Hazard ratio
IMRT: Intensity-modulated radiotherapy
LC: Local control
LN: Lymph node
OARs: Organs at risk
OS: Overall survival
PET-CT: Positron emission tomography-computed tomography
PFS: Progression-free survival
PR: Partial response
PTV: Planning target volume
SABR: Stereotactic ablative radiotherapy
SBRT: Stereotactic body radiotherapy
