# Supplementary material for: Role of high-dose salvage radiotherapy for oligometastases of the localised abdominal/pelvic lymph nodes: a retrospective study
Source: BMC Cancer. 2020 Jun 9;20:540. doi: 10.1186/s12885-020-07033-7 (PMC7285737; doi:10.1186/s12885-020-07033-7)
Supplement: Supplementary file 1 — Additional file 1: Supplementary Table 1. Treatment details for each patient. Supplementary Table 2. Number of lymph node metastases by site. Supplementary Table 3. Patient characteristics in the solitary oligometastasis group. [file 12885_2020_7033_MOESM1_ESM.docx]

Supplementary Table 1. Treatment details for each patient.

| Patients | Modality | Technique | Dose/ fraction (Gy) | Fractions | Prescription dose before standardization (Gy) | Prescription dose (D50) | EQD2 (D50) |
| --- | --- | --- | --- | --- | --- | --- | --- |
| 1 | Linear accelerator | IMRT | 2 | 33 | 66 | 67.6 | 67.9 |
| 2 | Linear accelerator | IMRT | 2.6 | 25 | 65 | 65.0 | 68.3 |
| 3 | Linear accelerator | IMRT | 2.6 | 20 | 52 | 52.7 | 55.5 |
| 4 | Linear accelerator | IMRT | 1.8 | 28 | 50.4 | 51.3 | 50.6 |
| 5 | Linear accelerator | IMRT | 2 | 30 | 60 | 62.2 | 62.6 |
| 6 | Linear accelerator | IMRT | 3.5 | 15 | 52.5 | 54.2 | 61.5 |
| 7 | Linear accelerator | IMRT | 2 | 30 | 60 | 61.6 | 61.9 |
| 8 | Linear accelerator | IMRT | 2 | 26 | 52 | 53.4 | 53.6 |
| 9 | Linear accelerator | IMRT | 2.5 | 25 | 62.5 | 63.7 | 66.6 |
| 10 | Linear accelerator | IMRT | 2.5 | 20 | 50 | 49.3 | 51.2 |
| 11 | Linear accelerator | IMRT | 3.5 | 20 | 70 | 71.4 | 80.7 |
| 12 | Linear accelerator | IMRT | 2 | 35 | 70 | 71.9 | 72.2 |
| 13 | Linear accelerator | 3DCRT | 1.8 | 34 | 61.2 | 60.2 | 59.0 |
| 14 | Linear accelerator | 3DCRT | 2 | 35 | 70 | 64.8 | 64.0 |
| 15 | Linear accelerator | 3DCRT | 1.8 | 26 | 46.8 | 47.6 | 46.9 |
| 16 | Linear accelerator | 3DCRT | 2 | 30 | 60 | 52.0 | 50.8 |
| 17 | Linear accelerator | 3DCRT | 2 | 23 | 46 | 45.6 | 45.5 |
| 18 | Linear accelerator | 3DCRT | 2 | 25 | 50 | 49.6 | 49.5 |
| 19 | Linear accelerator | 3DCRT | 2 | 25 | 50 | 49.8 | 49.8 |
| 20 | Linear accelerator | 3DCRT | 2 | 23 | 46 | 45.5 | 45.4 |
| 21 | Linear accelerator | 3DCRT | 1.8 | 28 | 50.4 | 51.5 | 50.8 |
| 22 | Linear accelerator | 3DCRT | 2 | 30 | 60 | 60.5 | 60.6 |
| 23 | Linear accelerator | 3DCRT | 2 | 25 | 50 | 46.9 | 46.4 |
| 24 | Linear accelerator | 3DCRT | 2 | 32 | 64 | 65.8 | 66.1 |
| 25 | Linear accelerator | 3DCRT | 1.8 | 36 | 64.8 | 62.6 | 61.2 |
| 26 | Linear accelerator | 3DCRT | 2 | 35 | 70 | 71.2 | 71.4 |
| 27 | Linear accelerator | 3DCRT | 2 | 32 | 64 | 66.5 | 66.9 |
| 28 | Linear accelerator | 3DCRT | 1.8 | 37 | 66.6 | 66.5 | 65.4 |
| 29 | Linear accelerator | 3DCRT | 2 | 35 | 70 | 65.5 | 64.8 |
| 30 | Linear accelerator | 3DCRT | 2 | 32 | 64 | 65.2 | 65.4 |
| 31 | Linear accelerator | 3DCRT | 1.8 | 33 | 59.4 | 58.3 | 57.2 |
| 32 | Linear accelerator | 3DCRT | 2 | 25 | 50 | 48.8 | 48.6 |
| 33 | Linear accelerator | 3DCRT | 2 | 35 | 70 | 69.3 | 69.2 |
| 34 | Linear accelerator | 3DCRT | 2 | 25 | 50 | 49.8 | 49.8 |
| 35 | Linear accelerator | 3DCRT | 2.2 | 22 | 48.4 | 48.4 | 49.2 |
| 36 | Linear accelerator | 3DCRT | 2 | 25 | 50 | 50.0 | 50.0 |
| 37 | Linear accelerator | 3DCRT | 3 | 15 | 45 | 45.0 | 48.8 |
| 38 | Linear accelerator | 3DCRT | 2 | 25 | 50 | 50.0 | 50.0 |
| 39 | Linear accelerator | 3DCRT | 2 | 25 | 50 | 50.0 | 50.0 |
| 40 | Linear accelerator | 3DCRT | 2.5 | 19 | 47.5 | 42.5 | 43.3 |
| 41 | Linear accelerator | 3DCRT | 2.5 | 15 | 37.5 | 38.5 | 40.3 |
| 42 | Linear accelerator | 3DCRT | 1.8 | 28 | 50.4 | 50.4 | 49.6 |
| 43 | Linear accelerator | 3DCRT | 2 | 25 | 50 | 50.0 | 50.0 |
| 44 | Linear accelerator | 3DCRT | 1.8 | 28 | 50.4 | 50.4 | 49.6 |
| 45 | Linear accelerator | 3DCRT | 2 | 30 | 60 | 59.5 | 59.4 |
| 46 | Linear accelerator | 3DCRT | 2 | 25 | 50 | 50.0 | 50.0 |
| 47 | Linear accelerator | 3DCRT | 2 | 25 | 50 | 49.4 | 49.3 |
| 48 | Linear accelerator | 3DCRT | 1.8 | 35 | 63 | 63.4 | 62.4 |
| 49 | Linear accelerator | 3DCRT | 2 | 30 | 60 | 59.8 | 59.8 |
| 50 | Linear accelerator | 3DCRT | 2 | 30 | 60 | 61.2 | 61.4 |
| 51 | Linear accelerator | 3DCRT | 1.8 | 22 | 39.6 | 41.0 | 40.5 |
| 52 | Linear accelerator | 3DCRT | 2 | 25 | 50 | 49.5 | 49.4 |
| 53 | Linear accelerator | 3DCRT | 2 | 25 | 50 | 49.5 | 49.4 |
| 54 | Linear accelerator | IMRT | 2 | 30 | 60 | 60.4 | 60.5 |
| 55 | Linear accelerator | 3DCRT | 2 | 25 | 50 | 50.5 | 50.6 |
| 56 | Linear accelerator | IMRT | 2.5 | 18 | 45 | 45.1 | 47.0 |
| 57 | Linear accelerator | 3DCRT | 2.5 | 16 | 40 | 39.5 | 41.0 |
| 58 | Linear accelerator | 3DCRT | 1.8 | 25 | 45 | 45.0 | 44.3 |
| 59 | Linear accelerator | 3DCRT | 1.8 | 28 | 50.4 | 50.0 | 49.1 |
| 60 | Linear accelerator | 3DCRT | 2 | 25 | 50 | 50.0 | 50.0 |
| 61 | Linear accelerator | 3DCRT | 2 | 25 | 50 | 50.0 | 50.0 |
| 62 | CyberKnife | SBRT | 6 | 8 | 48 | 51.2 | 70.0 |
| 63 | CyberKnife | IMRT | 2 | 25 | 50 | 54.8 | 55.7 |
| 64 | CyberKnife | IMRT | 2 | 25 | 50 | 52.0 | 52.3 |
| 65 | CyberKnife | SBRT | 5 | 10 | 50 | 56.5 | 73.7 |
| 66 | CyberKnife | SBRT | 5 | 10 | 50 | 55.5 | 71.9 |
| 67 | CyberKnife | IMRT | 2 | 30 | 60 | 62.5 | 62.9 |
| 68 | CyberKnife | SBRT | 9 | 3 | 27 | 37.5 | 70.3 |
| 69 | CyberKnife | SBRT | 5 | 7 | 35 | 41.0 | 54.2 |
| 70 | CyberKnife | SBRT | 6 | 5 | 30 | 37.1 | 53.9 |
| 71 | CyberKnife | SBRT | 13.5 | 2 | 27 | 35.0 | 80.2 |
| 72 | CyberKnife | SBRT | 7 | 3 | 21 | 31.2 | 53.0 |
| 73 | CyberKnife | SBRT | 10 | 3 | 30 | 38.6 | 73.6 |
| 74 | CyberKnife | SBRT | 9 | 3 | 27 | 41.2 | 81.5 |
| 75 | CyberKnife | SBRT | 5 | 8 | 40 | 51.0 | 69.6 |
| 76 | CyberKnife | SBRT | 6 | 5 | 30 | 36.9 | 53.4 |
| 77 | CyberKnife | SBRT | 5 | 5 | 25 | 36.4 | 52.4 |
| 78 | CyberKnife | SBRT | 6 | 5 | 30 | 39.9 | 59.8 |
| 79 | CyberKnife | SBRT | 4 | 7 | 28 | 55.0 | 81.8 |
| 80 | CyberKnife | SBRT | 6 | 5 | 30 | 38.7 | 57.2 |
| 81 | CyberKnife | SBRT | 10 | 3 | 30 | 37.1 | 69.2 |
| 82 | CyberKnife | SBRT | 7 | 5 | 35 | 42.9 | 66.4 |
| 83 | CyberKnife | SBRT | 9 | 3 | 27 | 32.7 | 57.0 |
| 84 | CyberKnife | SBRT | 6 | 5 | 30 | 41.7 | 63.7 |
| 85 | CyberKnife | SBRT | 6 | 5 | 30 | 41.1 | 62.4 |
| 86 | CyberKnife | SBRT | 8 | 3 | 24 | 46.8 | 99.8 |
| 87 | CyberKnife | SBRT | 5 | 5 | 25 | 31.0 | 41.9 |
| 88 | CyberKnife | SBRT | 7 | 5 | 35 | 41.6 | 63.5 |
| 89 | CyberKnife | SBRT | 7 | 5 | 35 | 42.5 | 65.5 |
| 90 | CyberKnife | SBRT | 7 | 5 | 35 | 42.0 | 64.4 |
| 91 | CyberKnife | SBRT | 10 | 3 | 30 | 34.6 | 62.1 |
| 92 | CyberKnife | SBRT | 9 | 3 | 27 | 32.0 | 55.1 |
| 93 | CyberKnife | SBRT | 6 | 5 | 30 | 37.0 | 53.7 |
| 94 | CyberKnife | SBRT | 6 | 5 | 30 | 40.6 | 61.3 |
| 95 | CyberKnife | SBRT | 7 | 5 | 35 | 40.2 | 60.4 |
| 96 | CyberKnife | SBRT | 5 | 6 | 30 | 35.8 | 47.6 |
| 97 | CyberKnife | SBRT | 5 | 7 | 35 | 44.9 | 61.4 |
| 98 | CyberKnife | SBRT | 7 | 5 | 35 | 41.6 | 63.5 |
| 99 | CyberKnife | SBRT | 7 | 5 | 35 | 46.2 | 74.1 |
| 100 | CyberKnife | SBRT | 10 | 3 | 30 | 39.4 | 76.0 |
| 101 | CyberKnife | IMRT | 2 | 30 | 60 | 63.5 | 64.1 |
| 102 | CyberKnife | SBRT | 7.25 | 8 | 58 | 66.5 | 101.5 |
| 103 | CyberKnife | IMRT | 3 | 20 | 60 | 73.0 | 83.0 |
| 104 | CyberKnife | SBRT | 5 | 10 | 50 | 55.0 | 71.0 |
| 105 | CyberKnife | IMRT | 2 | 25 | 50 | 54.0 | 54.7 |
| 106 | CyberKnife | IMRT | 2 | 30 | 60 | 62.5 | 62.9 |
| 107 | CyberKnife | SBRT | 10 | 5 | 50 | 54.5 | 94.9 |
| 108 | CyberKnife | SBRT | 5 | 10 | 50 | 54.0 | 69.3 |
| 109 | CyberKnife | SBRT | 4.2 | 10 | 42 | 45.3 | 54.9 |
| 110 | CyberKnife | IMRT | 3 | 20 | 60 | 63.5 | 69.7 |
| 111 | CyberKnife | IMRT | 2 | 25 | 50 | 53.5 | 54.1 |
| 112 | CyberKnife | IMRT | 2 | 25 | 50 | 52.5 | 52.9 |
| 113 | CyberKnife | SBRT | 10 | 3 | 30 | 38.3 | 72.7 |
|  |  |  |  |  |  |  |  |
| 3DCRT, 3-dimentional conformal radiation therapy; SBRT, Stereotactic Body Radiotherapy; IMRT, intensity-modulated radiotherapy; EQD2, equivalent dose in 2 Gy fraction; D50, dose to 50 % of the volume. | | | | | | |  |
|  |  |  |  |  |  |  |  |
|  |  |  |  |  |  |  |  |

Supplementary Table 2. Number of lymph node metastases by site.

|  |  | Number of lymph node | | | | |
| --- | --- | --- | --- | --- | --- | --- |
|  |  | Solitary (n=61) | Two (n=20) | Three (n=19) | Four (n=6) | Five (n=7) |
| Lymph node site | Para-aortic | 40 | 11 | 12 | 4 | 5 |
|  | Iliac | 10 | 3 | 2 | 1 | 2 |
|  | Presacral | 6 | 4 | 1 | 1 | 0 |
|  | Obturator | 5 | 2 | 4 | 0 | 0 |

Supplementary Table 3.Patient characteristics in the solitary oligometastasis group.

| Characteristic | | High-dose  group (n=33) | Low-dose  group（n=28） |
| --- | --- | --- | --- |
| Initial category | T-category 1:2:3:4 | 11:10:11:1 | 6:8:11:3 |
|  | N-category positive | 17 | 13 |
| DFI (months) | Median | 8.7 | 9.5 |
|  | Range | 0.6-64.4 | 0.5-86.6 |
| Extra-regional LN for primary site | yes | 11 | 12 |
| Follow-up time in  surviving patients (months) | Median | 22.7 | 23.8 |
|  | Range | 7.6-84.7 | 4.0-60.0 |
|  |  |  |  |
| n, number of patients; DFI, disease-free interval; LN, lymph node. | | | |
